# Oxidation of benzoin catalyzed by oxovanadium(IV) schiff base complexes

**DOI:** 10.1186/1752-153X-7-3

**Published:** 2013-01-07

**Authors:** Tahseen A Alsalim, Jabbar S Hadi, Omar N Ali, Hanna S Abbo, Salam JJ Titinchi

**Affiliations:** 1 Department chemistry, College of Education, University of Basrah, Basrah, Iraq; 2 Department of Chemistry, University of the Western Cape Bellville, Private Bag X17, , Bellville, Cape Town 7535, South Africa

**Keywords:** Tridentate ONO Schiff base ligands, Oxovanadium(IV) complexes, Benzoin oxidation, Benzil

## Abstract

**Background:**

The oxidative transformation of benzoin to benzil has been accomplished by the use of a wide variety of reagents or catalysts and different reaction procedures. The conventional oxidizing agents yielded mainly benzaldehyde or/and benzoic acid and only a trace amount of benzil. The limits of practical utilization of these reagents involves the use of stoichiometric amounts of corrosive acids or toxic metallic reagents, which in turn produce undesirable waste materials and required high reaction temperatures.

In recent years, vanadium complexes have attracted much attention for their potential utility as catalysts for various types of reactions.

**Results:**

Active and selective catalytic systems of new unsymmetrical oxovanadium(IV) Schiff base complexes for the oxidation of benzoin is reported. The Schiff base ligands are derived between 2-aminoethanol and 2-hydroxy-1-naphthaldehyde (H_2_L^1^) or 3-ethoxy salicylaldehyde (H_2_L^3^); and 2-aminophenol and 3-ethoxysalicylaldehyde (H_2_L^2^) or 2-hydroxy-1-naphthaldehyde (H_2_L^4^). The unsymmetrical Schiff bases behave as tridentate dibasic ONO donor ligands. Reaction of these Schiff base ligands with oxovanadyl sulphate afforded the mononuclear oxovanadium(IV) complexes (V^IV^OL^x^.H_2_O), which are characterized by various physico-chemical techniques.

The catalytic oxidation activities of these complexes for benzoin were evaluated using H_2_O_2_ as an oxidant. The best reaction conditions are obtained by considering the effect of solvent, reaction time and temperature. Under the optimized reaction conditions, VOL^4^ catalyst showed high conversion (>99%) with excellent selectivity to benzil (~100%) in a shorter reaction time compared to the other catalysts considered.

**Conclusion:**

Four tridentate ONO type Schiff base ligands were synthesized. Complexation of these ligands with vanadyl(IV) sulphate leads to the formation of new oxovanadium(IV) complexes of type V^IV^OL.H_2_O.

Elemental analyses and spectral data of the free ligands and their oxovanadium(IV) complexes were found to be in good agreement with their structures, indicating high purity of all the compounds.

Oxovanadium complexes were screened for the oxidation of benzoin to benzil using H_2_O_2_ as oxidant. The effect of time, solvent and temperature were optimized to obtain maximum yield. The catalytic activity results demonstrate that these catalytic systems are both highly active and selective for the oxidation of benzoin under mild reaction conditions.

## Background

Liquid phase catalytic oxidation of alcohols is a fascinating reaction and is one of the most important synthetic reactions in organic chemistry. Oxidation of benzoin to benzil has been extensively studied for the production of fine chemicals [1-6]. In general α-dicarbonyl compounds are important synthetic intermediates in the synthesis of many heterocyclic compounds. α-Dicarbonyl compounds have diverse applications in organic and pharmaceutical industries such as photosensitive and synthetic reagents [7,8] and photo initiators for radical polymerization [9]. Benzil, in particular, is a standard building block in organic synthesis and is utilized as an intermediate in the synthesis of chiral ligands and biologically active compounds.

The oxidative transformation of an α-hydroxy ketone to the corresponding α-diketone (benzoin to benzil) has been accomplished by the use of a wide variety of reagents or catalysts and different reaction procedures. Several reagents have been used for this transformation such as nitric acid, Th(III) and Y(III) nitrate [10] and bismuth(III) nitrate-copper(II) acetate [11]. The conventional oxidizing agents viz*.*, permanganate, dichromate or chromic acid yielded chiefly benzaldehyde/benzoic acid and only a trace of benzil. The limits of practical utilization of these reagents involves the use of stoichiometric amounts of corrosive acids or toxic metallic reagents, which in turn produce undesirable waste materials and required high reaction temperatures.

In recent years, vanadium complexes have attracted much attention for their potential utility as catalysts for various types of reactions. Oxovanadium complexes catalyze various oxidation reactions viz*.*, hydroxylation of phenols [12,13], oxidation of sulfides to sulfoxides [14,15], oxidation of alcohols [15], epoxidation of olefins [15-19], hydroxylation of benzene to mono- and dihydroxybenzenes [12,20,21] including reactions such as the coupling of 2-naphthols as well as Mannich-type reactions [22-25]. Most recently, the application of various oxovanadium complexes as catalysts in different oxidation reactions has been reviewed [26,27].

In this report we describe the synthesis of Schiff base ligands derived by reaction between 2-aminophenol or 2-aminoethanol and 2-hydroxy-1-naphthaldehyde or 3-ethoxy salicylaldehyde to form tridentate dibasic ligands of the ONO type. Complexation of these ligands with vanadyl sulphate produced oxovanadium(IV) complexes. To the best of our knowledge, this is the first report describing the synthesis of these oxovanadium(IV) complexes.

As part of our continuing interest in oxidation reactions by vanadium complexes [13] and considering the demand of more efficient catalytic systems, we undertook an investigation of these oxovanadium(IV) complexes as catalysts for benzion oxidation for the first time.

## Results and discussion

### Synthesis and characterization

**Scheme 1:**
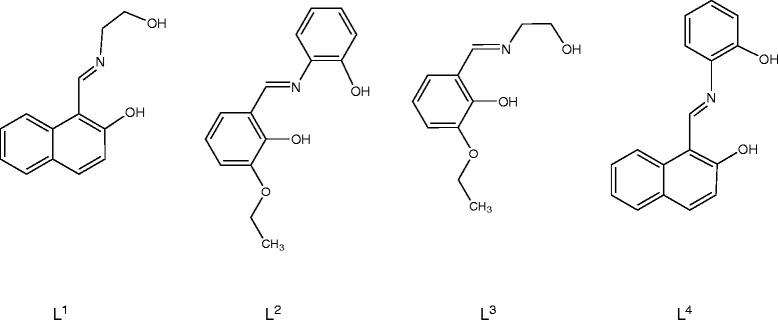
Structure of the ligands.

**Scheme 2:**
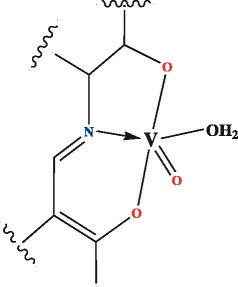
General structure of oxovanadium(IV) complexes.

Facile condensation of 2-aminophenol or 2-aminoethanol and 2-hydroxy-1-naphthaldehyde or 3-ethoxy salicylaldehyde in 1:1 molar ratio afforded four Schiff base ligands viz*.* H_2_L^1^, H_2_L^2^, H_2_L^3^ and H_2_L^4^, respectively (Scheme 1). These tridentate ligands reacted readily with vanadyl sulfate in methanol to form the oxovanadium complexes with the same general structure (Scheme 2).

Spectral data and elemental analysis of all the synthesized ligands and their oxovanadium(IV) complexes were in good agreement with their structure, indicating the high purity of all the compounds.

The analytical data of the complexes indicates a 1:1 metal: ligand stoichiometry, and hence the Schiff bases act as tridentate dibasic ligands. These ligands can coordinate through the imino nitrogen and two oxygen atoms from the deprotonation of the phenolic groups as well as from the aliphatic hydroxyl group. The ligands react with V(IV) to fill three of the four equatorial coordination sites with a water molecule coordinating to the remaining site.

The IR spectra of the ligands and their complexes were compared to determine any changes during complexation and to confirm their structures (Additional file 1: Figure S1-S3). The IR spectra of the ligands showed a broad absorption band at 3230–3117 cm^-1^ attributed to the stretching vibration of intramolecular hydrogen bonded OH groups [28]. This band disappeared on complexation indicating the coordination of vanadium metal through OH groups after deprotonation. A new medium intensity band near 3400–3500 cm^-1^ is attributed to the νOH stretching vibration of the coordinated water molecule to the central metal ion [29]. A strong band at 1648–1629 cm^-1^ of the spectra of the ligand was assigned to the azomethine (C=N) stretching vibration. This band shifted to lower frequency (by 10–30 cm^-1^) in the complexes, indicating that the nitrogen atom of the azomethine group is coordinated to the metal centre [30,31]. The phenolic ν(C–O) band at 1114–1116 cm^-1^ in the free ligand is shifted towards lower frequency by 10 ± 15 cm^-1^ in the complexes, verifying coordination via the deprotonated phenolic oxygen. The complexes show a new strong band in the region between 975–987 cm^-1^ assigned to stretching vibration of V=O [32,33] which indicates its monomeric nature [34]. The absence of significant bands in the frequency range below 900 cm^-1^ demonstrates no V=O····V bridge vibration [35,36]. A similar monomeric nature of related complexes has also been well established crystallographically [37,38]. The appearance of two to three new moderately intense bands in the low-frequency region of 350–500 cm^-1^ in the complexes are assigned to stretching frequencies of ν(V–N) and ν(V–O) bonds i.e. coordination of azomethine nitrogen as well as phenolic oxygen to the vanadium metal after deprotonation. The C=C stretching modes of the benzene ring of the ligand around 1600 cm^-1^ does not show any significant shift on complexation. Thus, the IR data indicates that the Schiff bases behave as dibasic tridentate ligands coordinating through phenolic and alcoholic oxygens and the azomethine nitrogen.

The ^1^H NMR spectra of ligand H_2_L^1^ and ligand H_2_L^3^ displayed a broad signal corresponding to the OH proton of the ethanol amine moiety at 5.00 and 5.32 ppm, respectively. The phenolic OH protons appeared as broad signals at 13.88 and 13.70 ppm, respectively. The ^1^H NMR spectra of ligands H_2_L^2^ and H_2_L^4^ displayed the OH phenolic groups as singlets at 9.77 and 14.2, and 10.35 and 15.7 ppm, respectively [39]. In all the ligands the azomethine HC=N proton appeared as a singlets at 8.35−9.43 ppm. All the other aromatic and aliphatic protons were observed in the expected regions (Additional file 1: Figure S4-S7).

The elemental analyses of the ligands and the complexes are in agreement with their formulation. The elemental analysis of the complexes confirms a 1:1 (metal: ligand) stiochiometry.

The result of mass spectra further indicates that the V(IV) complexes have a monomeric form of 1:1 stiochiometry, where the observed molecular ion peak (m/z) values are consistent with the calculated value in the proposed structure (Additional file 1: Figure S8-S11). The complexes were non-volatile and difficulties were experienced to record their mass spectra by E1 methods.

### Catalytic activity studies

The catalytic activity of oxovanadium complexes for the oxidation of benzoin to benzil has been studied and the effect of time, solvent and temperature were optimized to produce maximum yield. Oxidation did not proceed when the reaction was carried out in the presence of either hydrogen peroxide or oxovanadium complexes only.

Progress of the reaction (as determined by the concentration of benzil) was monitored spectrophotometrically and identification of the product was confirmed by GC-MS analyses which showed that benzil was the only product detectable. No oxidative cleavage products were observed.

**Figure 1:**
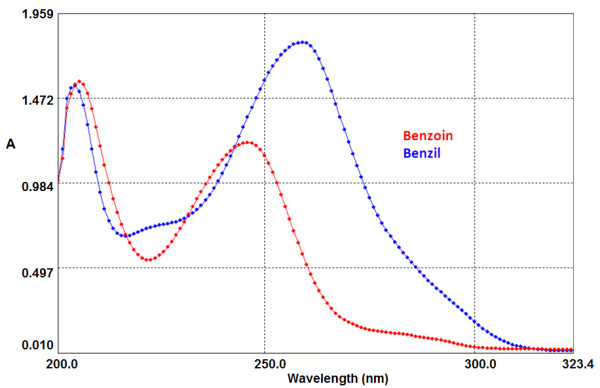
Absorption spectra of benzoin and benzil in acetonitrile.

The electronic spectrum of benzil is characterized by an absorption at 283 nm, which is readily differentiated from the other absorption bands of benzil at 260 nm and benzoin 247 nm [40]. Accordingly, the band at 283 nm was used to determine the concentration of the produced benzil (Figure 1).

In order to screen the catalytic oxidative potential of the prepared complexes, they were tested for oxidation of benzoin using H_2_O_2_ as an oxidant under different reaction conditions viz*.,* temperature, reaction time and type of solvent in order to optimise the conditions for the best performance of the catalyst.

**Figure 2:**
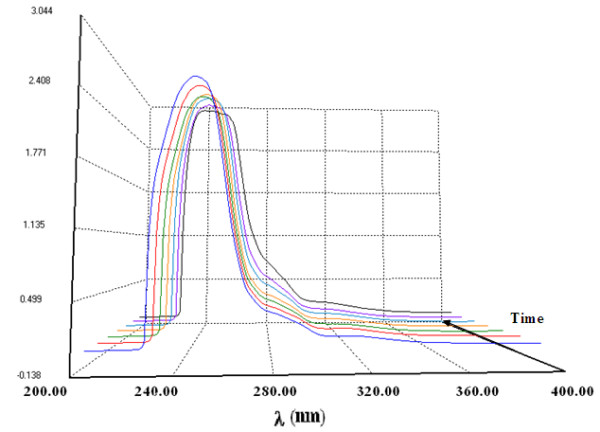
**Change in the absorption spectra with time using VOL**^**1**^**.**

**Figure 3:**
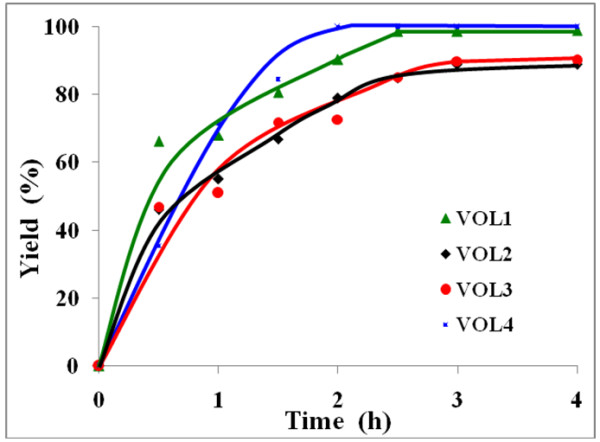
Catalytic performance of the different catalysts with time for the formation of benzil in benzene at 50°C.

Figure 2 shows that the absorbance at 283 nm increases with reaction time as an indication of the increasing concentration of benzil by using one of the catalysts, namely VOL^1^. The maximum benzil yield was found to depend upon the type of catalyst used (Figure 3). It is clear from Figure 3 that VOL^4^ and VOL^1^ give higher benzil yields compared to VOL^3^ and VOL^2^ with the same reaction time. VOL^4^ gave 100% yield after 2 h, while VOL^1^ gave 98.9% after a longer reaction time (4 h). The order of catalytic activity was found to be as follows: VOL^4^ > VOL^1^ > VOL^3^ >VOL^2^.

#### Effect of solvent

The influence of three different solvents viz*.* benzene, carbon tetrachloride and acetonitrile on the rate of benzoin oxidation was next studied.

**Figure 4:**
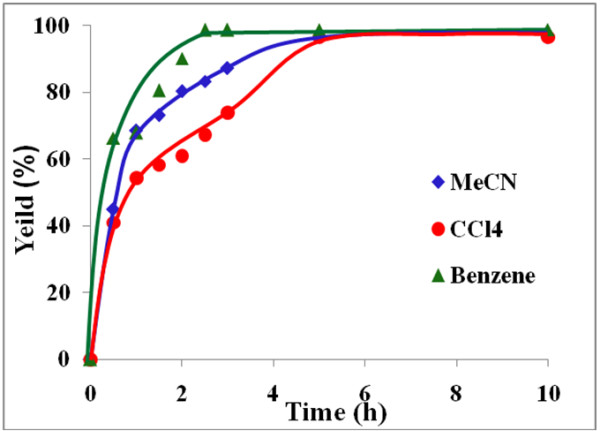
**Effect of solvent on % yield of benzil using VOL**^**1 **^**at 50°C.**

**Figure 5:**
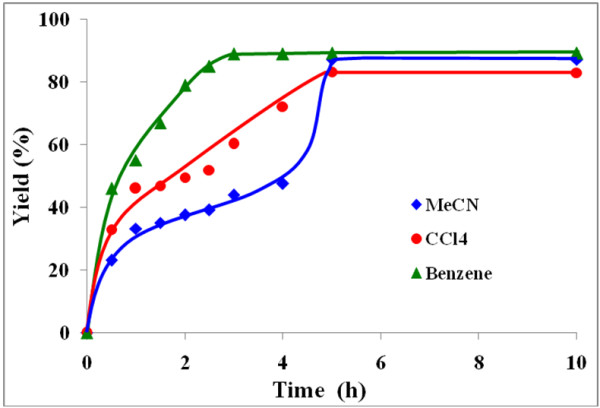
**Effect of solvent on % yield of benzil using VOL**^**2 **^**at 50°C.**

**Figure 6:**
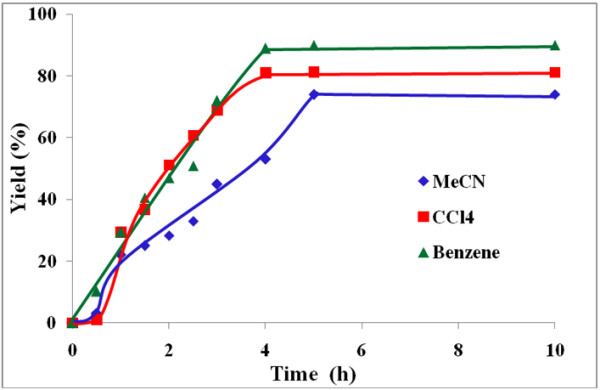
**Effect of solvent on % yield of benzil using VOL**^**3 **^**at 50°C.**

**Figure 7:**
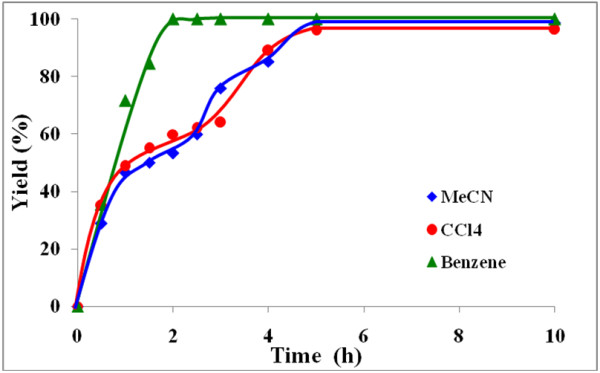
**Effect of solvent on % yield of benzil using VOL**^**4 **^**at 50°C.**

Figures 4, 5, 6, 7 illustrate the effect of solvent upon the rate and yield of benzoin using catalysts (VOL^1^−VOL^4^). It is clear from the figures that the solvent has no significant effect on the yield and selectivity. However, the reaction rate during the first four hours increases slightly as the polarity of the solvent decreases. Lower reaction rates were observed using the polar aprotic solvent, acetonitrile, compared to the other two solvents. On the other hand, the protic solvent, methanol, was not tried as it retards the oxidation of benzoin due to hydrogen bonding with the methanol [41].

In general, in these solvents, the order of the catalytic performance of these catalysts after 6 h reaction time as the following VOL^1^ ≅ VOL^4^ > VOL^3^ > VOL^2^.

#### Effect of temperature

**Figure 8:**
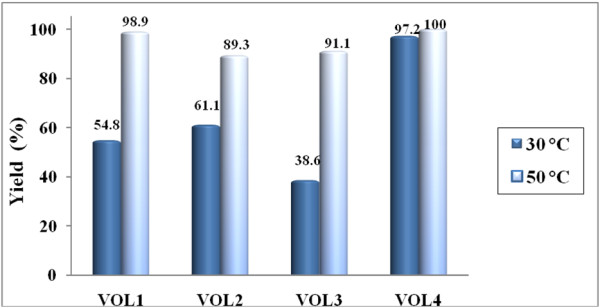
Catalytic performance of the catalysts for the oxidation of benzoin at two reaction temperatures in benzene after 5 h reaction time.

The performance of the catalysts was investigated at two different temperatures viz. 30°C and 50°C in benzene after 5 h reaction time (Figure 8).

In general, the catalytic activity of the four catalysts increased with increasing temperature. Figure 8 shows that there was a significant increase in benzil yield using VOL^1^, VOL^2^ and VOL^3^ when the reaction temperature was increased from 30°C to 50°C; while VOL^4^ gave a high benzil yield (97%) even at the lower temperature i.e. 30°C and a minor increase at 50°C. At 30°C, moderate yields of benzil were observed in all three solvents. However, the increase in reaction temperature to 50°C led to almost complete conversion of benzoin to benzil. Excellent selectivity was observed with all the catalysts. Thus from the results above it is clear that 50°C is the optimum temperature for this oxidation. All the catalysts possess excellent selectivity towards benzil formation.

#### Effect of time

The catalytic oxidation of benzoin using H_2_O_2_ as oxidant in the presence of the four catalysts was followed as a function of time in benzene at 50°C. The reaction profiles (Figure 3) showed that the yield of benzil increased with increasing reaction time until a steady state was reached after 2-4 h. However, maximum yield of benzil (>99.9%) was obtained using VOL^1^ and VOL^4^, and ca. 90% using VOL^2^ and VOL^3^. VOL^4^ achieved the maximum conversion within a shorter reaction time than the other catalysts. The selectivity of all the catalysts was found to be unaffected with increasing reaction time i.e. >99.9% benzil.

### Experimental section

#### Materials

Oxavanadium sulfate trihydrate, 2-aminophenol and 2-hydroxy-1-naphthaldehyde were obtained from Fluka, 2-aminoethanol, 3-ethoxysalicylaldehyde and benzoin were obtained from Merck 30% H_2_O_2_. All other chemical reagents were used as such. Solvents were used after being purified according to the standard methods.

### Physical methods and analysis

Infrared spectra were recorded as KBr pellets on a BUCK-500 FT-IR spectrometer. ^1^H NMR spectra were recorded on a Bruker 500 (500 MHz) spectrometer using DMSO-d_6_ as a solvent and TMS as internal standard. GC-MS were recorded on a Hewlett Packard E1 mass spectrometer at 70 eV. Elemental analysis was performed on Euro Vectro EA 3000A analyzer. All catalyzed reaction products were analyzed using UV–vis PG Instrument T-80^+^.

### Preparation methods

#### General method for preparation of Schiff base ligands

Methanolic solutions of the aldehyde and amine were mixed at room temperature in a 1: 1 molar ratio using glacial acetic acid as a catalytic agent. The resulting mixture was refluxed for 2 h. The crystalline product was obtained from the deep-yellow coloured solution after standing for a few hours at 25°C. The precipitate was filtered off and washed with ice-cold methanol. The pure compound was obtained by recrystallization from a suitable solvent.

H_2_L^1^ 1-{[(2-hydroxyethyl)imino]methyl}-2-naphthol

2-Hydroxy-1-naphthaldehyde (1.72 g), 2-aminoethanol (0.61 g), (69% yield) as orange needles (from ethanol); mp 148–150°C. *Anal.* Found: C, 71.69; H, 5.73; N, 6.09. C_13_H_13_NO_2_ requires C, 72.54; H, 6.09; N, 6.51%. IR (KBr, cm^-1^), 3167br (νOH), 3056 (νC-H Ar), 2917 (νC-H aliph.), 1641 (νC=N); ^1^H NMR (DMSO-d_6_, δ/ppm): 3.65 (m, 4 H, 2CH_2_), 5.00 (br, 1H, OH), 6.64-8.00 (m, 6H, Ar-H), 9.02(s, 1H, HC=N), 13.88 (br, 1H, phenolic OH); m/z M^.+^ = 215 (85%), 214 (66%), 184 (base peak), 170 (42%). Found 215.00, C_13_H_13_NO_2_ requires 215.25.

H_2_L^2^ 2-ethoxy-6-{[(2-hydroxyphenyl)imino]methyl}phenol

3-Ethoxysalicylaldehyde (1.66 g), 2-aminophenol (1.09 g), (87% yield) as reddish orange needles (from ethanol); mp 172-174°C. *Anal.* Found: C, 69.17; H, 5.41; N, 5.13. C_15_H_15_NO_3_ requires C, 70.02; H, 5.88; N, 5.44%. IR (KBr, cm^-1^), 32100 (νOH), 3060 (νC-H Ar), 2986 (νC-H aliph.), 1629 s(νC=N). ^1^H NMR (DMSO-d_6_, δ/ppm): 1.3 (t, 3H, CH_3_), 4.06 (q, 2H, 2CH_2_), 6.78-7.38 (m, 7H, Ar-H), 8.94 (s, 1H, HC=N), 9.77 (s, 1H, OH; amino phenol moiety), 14.20 (s, 1H, OH; 3-ethoxysalicylaldehyde moiety). m/z M^.+^ = 257 (86%), 242 (41%) [M-CH_3_]^.+^, 212 (24%) [M-OCH_2_CH_3_]^.+^, 120 (55%), 69 (base peak). Found 257.00, C_15_H_15_NO_3_ requires 257.29.

H_2_L^3^ 2-ethoxy-6-{[(2-hydroxyethyl)imino]methyl}phenol

3-Ethoxysalicylaldehyde (1.66 g), 2-aminoethanol (0.61 g), (81% yield) as yellow crystals (from ethanol); mp 88-90°C. *Anal.* Found: C, 62.25; H, 6.91; N, 6.41. C_11_H_15_NO_3_ requires C, 63.14; H, 7.23; N, 6.69%. IR (KBr, cm^-1^), 3230 (νOH), 3050 (νC-H Ar), 2953–2866 (νC-H aliph.), 1652 (νC=N). ^1^H NMR (DMSO-d_6_, δ/ppm): 1.6 (t, 3H, CH_3_), 3.70 (t, 2H, CH_2_ of CH_2_N=CH), 3.90 (t, 2H, CH_2_ of CH_2_OH), 5.32 (s, 1H, OH), 6.70-7.91 (m, 3H, Ar-H), 8.35(s, 1H, HC=N), 13.70 (br, 1H, OH phenolic OH); m/z M^.+^ = 209 (13%) C_11_H_15_NO_3_, 194 (20%) [M-CH_3_]^.+^, 164 (25%) [M-OCH_2_CH_3_]^.+^, 69 (base peak) (C_2_H_5_NO^+^). Found 209.00, C_11_H_15_NO_3_ requires 209.23.

H_2_L^4^ 1-{[(2-hydroxyphenyl)imino]methyl}-2-naphthol

2-Hydroxy-1-naphthaldehyde (1.72 g), 2-aminophenol (1.09 g), (63% yield) as orange needles (from ethanol); mp 247-249°C. *Anal.* Found: C, 76.64; H, 4.34; N, 4.96. C_17_H_13_NO_2_ requires C, 77.55; H, 4.98; N, 5.32%. IR (KBr, cm^-1^), 3117 (νOH), 3053 (νC-H Ar), 2960 (νC-H aliph.), 1622 (νC=N). ^1^H NMR (DMSO-d_6_, δ/ppm): 6.74-8.35 (m, 10H, Ar-H), 9.43 (s, 1H, HC=N), 10.35 (s, 1H, OH phenolic), 15.70 (s, 1H, OH naphthoyl); m/z M^.+^ = 263 (75%), 262 (base peak) [M-H]^.+^. Found 263.00. C_17_H_13_NO_2_ requires 263.30.

#### Preparation of the oxavanadium (IV) complexes

All complexes were prepared according to the following procedure: a hot methanolic solution of VOSO_4_.3H_2_O (0.217 g, 1 mmol), was added drop wise to a hot methanolic solution of the ligand (1.5 mmol). The resulting mixture was stirred for 3 h and the products obtained were filtered, washed with hot water then methanol and dried in air at 90°C.

VOL^1^ Dark green ppt., mp>300°C, IR (KBr, cm^-1^); 3050 (νC-H arom.), 2936 (νC-H aliph.) 1614 (νC=N), 1512 (νC=C), 1322 (νC-N), 1198 (ν-O), 975 (νV=O). *Anal.* Calcd. for C_13_H_13_NO_4_V: C, 52.35; H, 4.36; N, 4.69. Found: C, 52.51; H, 4.23; N, 4.51. m/z M^.+^ = 298.

VOL^2^ Brown ppt., mp>300°C, IR (KBr, cm^-1^); 3053 (νC-H arom.), 2969–2890 (νC-H aliph.), 1601 (νC=N), 1582 (νC=C), 1294 (νC-N), 1248 (νC-O), 987 (νV=O). *Anal.* calcd. for C_15_H_15_NO_5_V: C, 52.95; H, 4.41; N, 4.11. Found: C, 52.51; H, 4.23; N, 4.39. m/z M^.+^ = 340.

VOL^3^ Green ppt., mp>300°C, IR (KBr,cm^-1^); 3080 (υC-H arom.), 2951 (υC-H aliph.), 1608 (νC=N), 1505 (νC=C), 1334 (νC-N), 1212 (νC-O), 981 (νV=O). *Anal.* calcd. for C_11_H_15_NO_5_V: C, 45.21; H, 5.31; N, 4.79. Found: C, 45.54; H, 5.23; N, 4.57. m/z M^.+^ = 292.

VOL^4^ Dark green ppt., mp>300°C, IR (KBr,cm^-1^); 3067 (νC-H arom.), 2961 (νC-H aliph.), 1592 (νC=N), 1575 (νC=C), 1338 (νC-N), 1177 (νC-O), 978 (νV=O). *Anal.* calcd . for C_17_H_13_NO_4_V: C, 58.96; H, 3.75; N, 4.04. Found: C, 58.51; H, 3.49; N, 4.19. m/z M^.+^ = 346.

#### Catalytic activity study

The catalytic oxidation of benzoin to benzil was carried out in a 25 ml flask. In a typical reaction, an aqueous solution of 30% H_2_O_2_ (0.015 mmol) (0.5 mL of H_2_O_2_ stock solution; 0.017 g in 5 mL CH_3_CN) and benzoin (0.01 g, 0.047 mmol) were mixed in 3 mL of the solvent used and the reaction mixture was magnetically stirred and heated at 30°C or 50°C in an oil bath. An appropriate amount of catalyst (0.003 g) was added to the reaction mixture and with this, the reaction was considered to begin. Each run was repeated twice. During the reaction, the products were analysed after specific time intervals using UV–vis spectroscopy and later confirmed by GC after considering the response factors of the authentic samples. Samples of the reaction mixture (0.1 ml) were diluted with 10 ml mixture of H_2_O:CH_3_CN (1:1) and the absorbance of the solution was measured spectrophotometrically The effects of various parameters, such as time of reaction and type of solvent as well as the temperature of the reaction were studied in order to examine their effect on the reaction product pattern.

## Conclusion

Four tridentate ONO type Schiff base ligands were synthesized by condensation of 2-aminophenol or 2-aminoethanol with 3-ethoxy salicylaldehyde and 2-hydroxy-1-naphthaldehyde. Complexation of these ligands with vanadyl(IV) sulphate leads to the formation of new oxovanadium(IV) complexes of type V^IV^OL.H_2_O.

Elemental analyses and spectral data of the free ligands and their oxovanadium(IV) complexes were found to be in good agreement with their structures, indicating high purity of all the compounds.

Oxovanadium complexes were screened for the oxidation of benzoin to benzil using H_2_O_2_ as oxidant. The effect of time, solvent and temperature were optimized to obtain maximum yield. The catalytic activity results demonstrate that these catalytic systems are both highly active and selective for the oxidation of benzoin under mild reaction conditions.

## Competing interests

The authors declare that they have no competing interests.

## Authors’ contributions

OA carried out the experimental work. TA helped in experimental and discussion part. JH helped in experimental and discussion part. HA helped in the discussion, characterization and draft the manuscript. ST helped in the discussion, characterization and draft the manuscript. All authors read and approved the final manuscript.

## Supplementary Material

Additional file 1: Figure S1 Ft-IR spectrum of H_2_L^1^. **Figure S2: **FT-IR spectrum H_2_L^2^.**Figure S3: **FT-IR spectrum VOL^2^. **Figure S4:** H-NMR spectrum H_2_L^1^. **Figure S5: **H-NMR spectrum H_2_L^2^. **Figure S6: **H-NMR spectrum H_2_L^3^. **Figure S7:** H-NMR spectrum H_2_L^4^. **Figure S8: **Mass spectra H_2_L^1^. **Figure S9: **Mass spectra H_2_L^2^. **Figure S10: **Mass spectra H_2_L^3^. **Figure S11: **Mass spectra H_2_L^4^. Click here for file
