# Supplementary material for: Oxidation of benzoin catalyzed by oxovanadium(IV) schiff base complexes
Source: Chem Cent J. 2013 Jan 7;7:3. doi: 10.1186/1752-153X-7-3 (PMC3599288; doi:10.1186/1752-153X-7-3)
Supplement: Additional file 1: Figure S1 — Ft-IR spectrum of H2L1. Figure S2: FT-IR spectrum H2L2.Figure S3: FT-IR spectrum VOL2. Figure S4: H-NMR spectrum H2L1. Figure S5: H-NMR spectrum H2L2. Figure S6: H-NMR spectrum H2L3. Figure S7: H-NMR spectrum H2L4. Figure S8: Mass spectra H2L1. Figure S9: Mass spectra H2L2. Figure S10: Mass spectra H2L3. Figure S11: Mass spectra H2L4. [file 1752-153X-7-3-S1.pdf]

L1

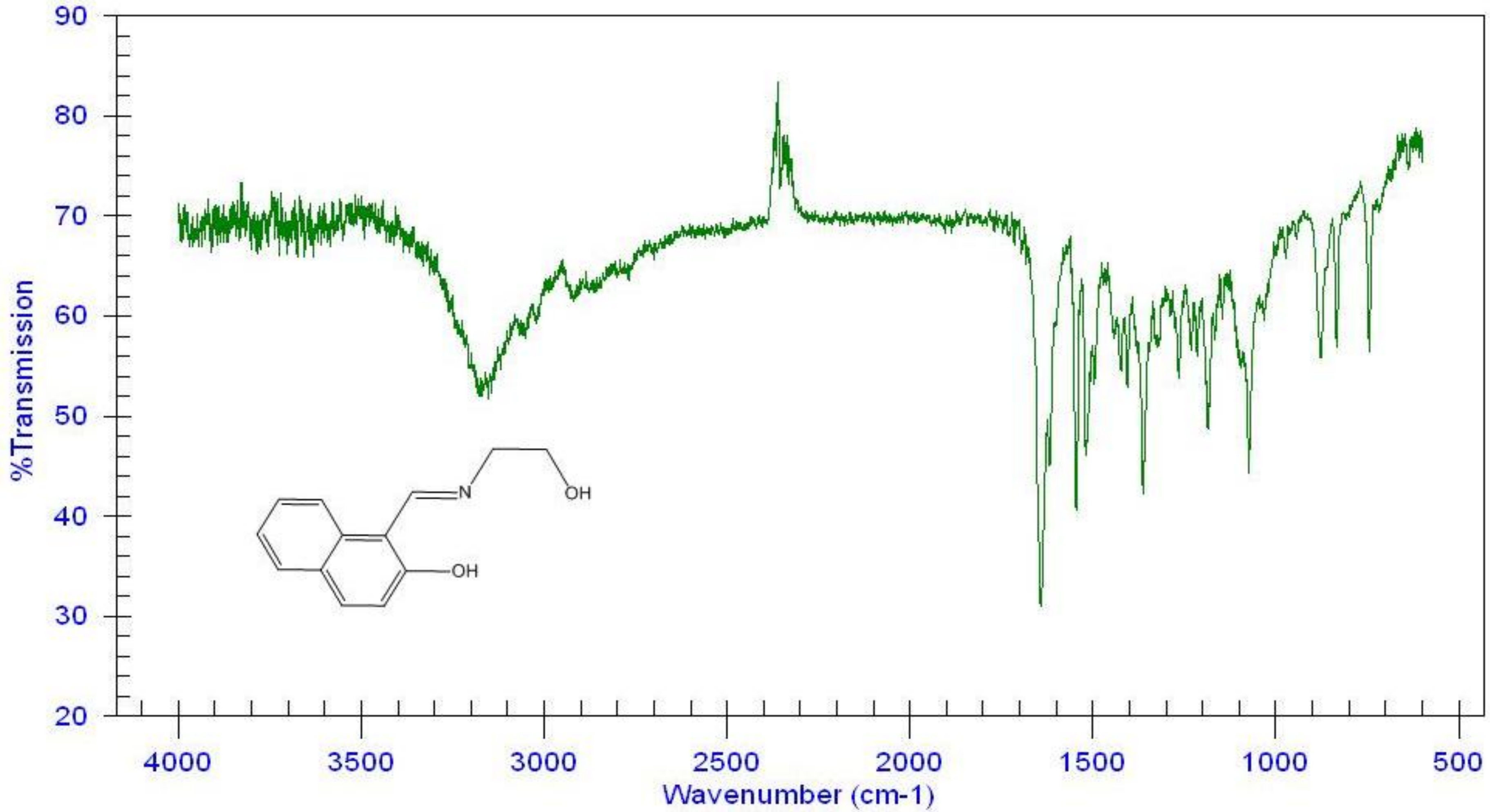
90
80
70
60
50
40
30
20
%Transmission
4000
3500
3000
2500
2000
1500
1000
500
Wavenumber (cm-1)
N
OH
OH

L2

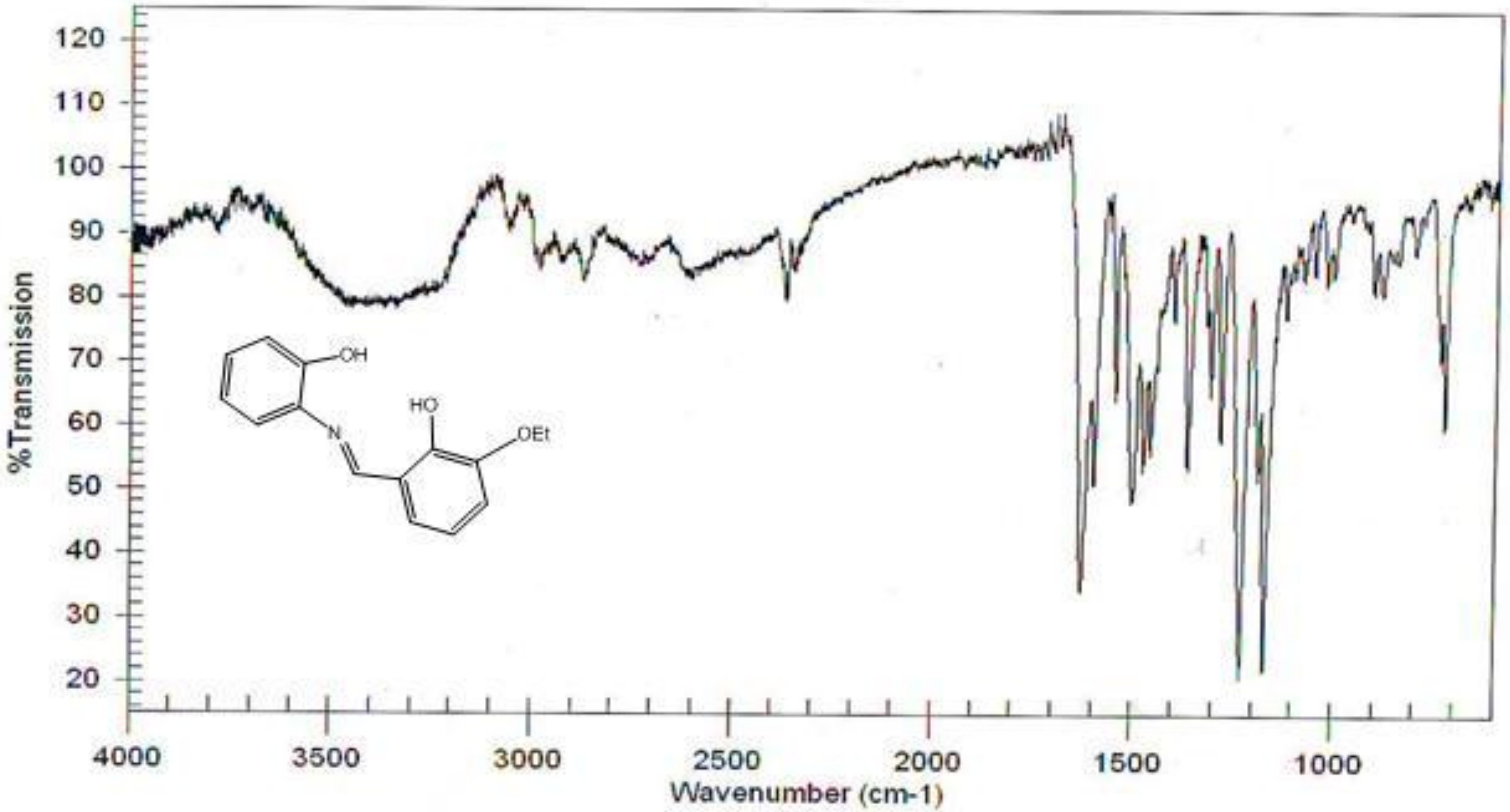
120
110
100
90
80
70
60
50
40
30
20
%Transmission
OH
HO
N
OEt
4000
3500
3000
2500
2000
1500
1000
Wavenumber (cm-1)

VOL2

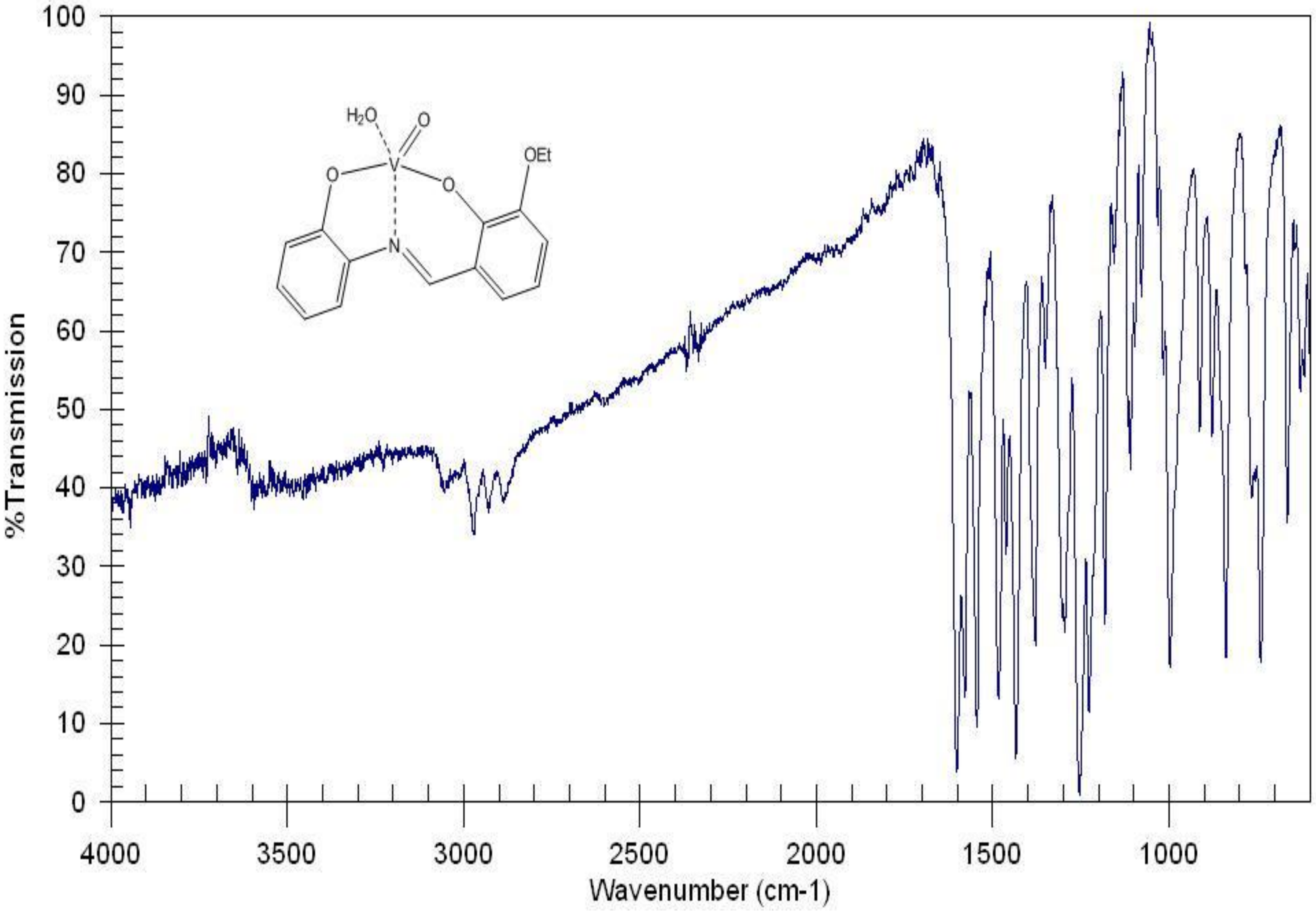
100
90
80
70
60
50
40
30
20
10
0
%Transmission
4000
3500
3000
2500
2000
1500
1000
Wavenumber (cm-1)
H2O
O
V
O
O
OEt
N

L1

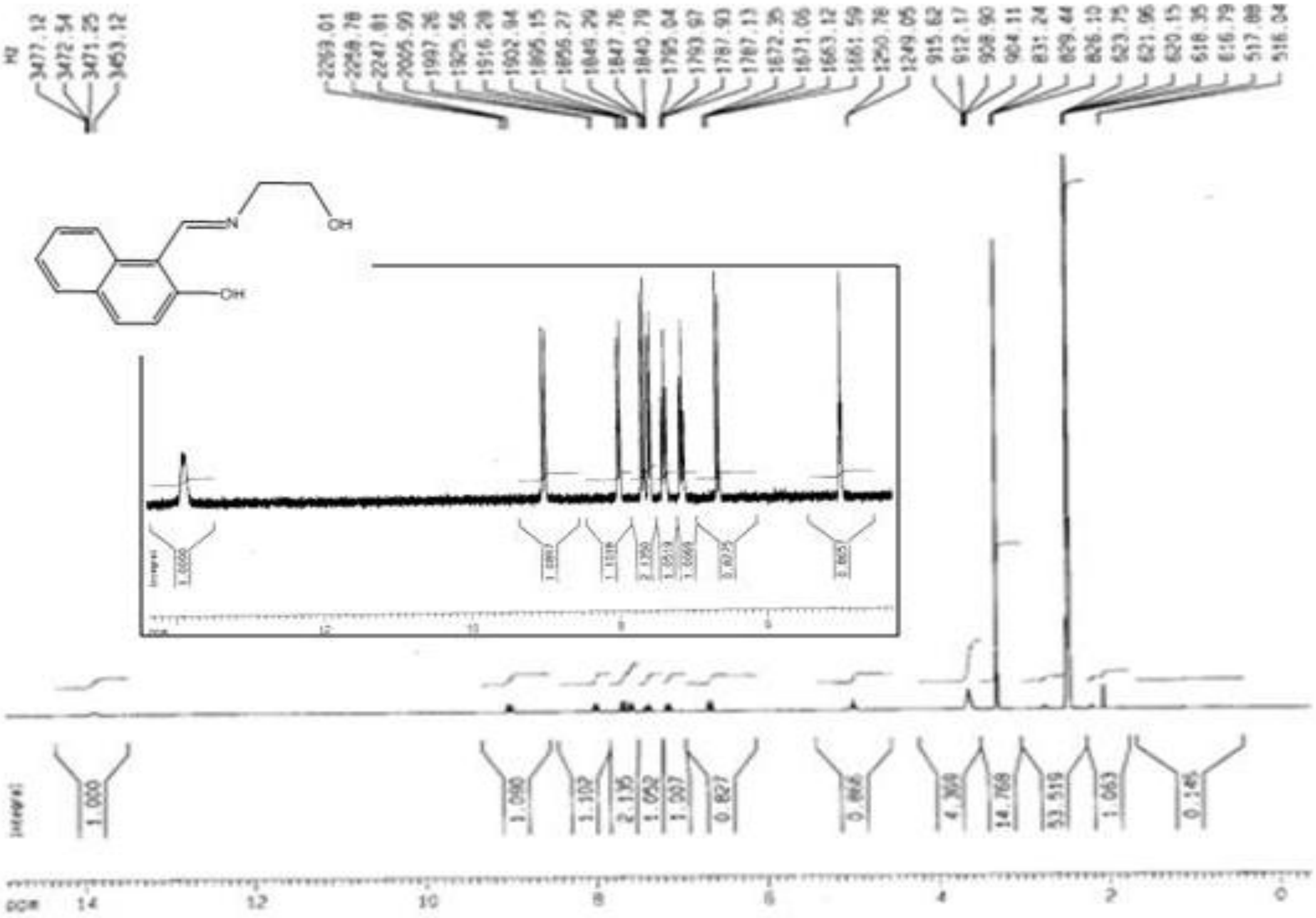
Hz
3477.12
3472.54
3471.25
3453.12
2259.01
2258.78
2247.81
2005.90
1997.26
1925.56
1916.28
1902.94
1895.15
1856.27
1849.29
1847.76
1840.79
1795.04
1793.97
1787.93
1787.13
1672.35
1671.06
1663.12
1661.59
1250.78
1249.05
915.62
912.17
908.90
904.11
831.24
829.44
826.10
623.75
621.96
620.15
618.35
616.79
517.88
516.04
OH
N
OH
Integral
1.000
1.090
1.102
2.135
1.052
1.007
0.927
0.866
4.368
14.768
53.519
1.063
0.145
ppm
14
12
10
8
6
4
2
0

L2

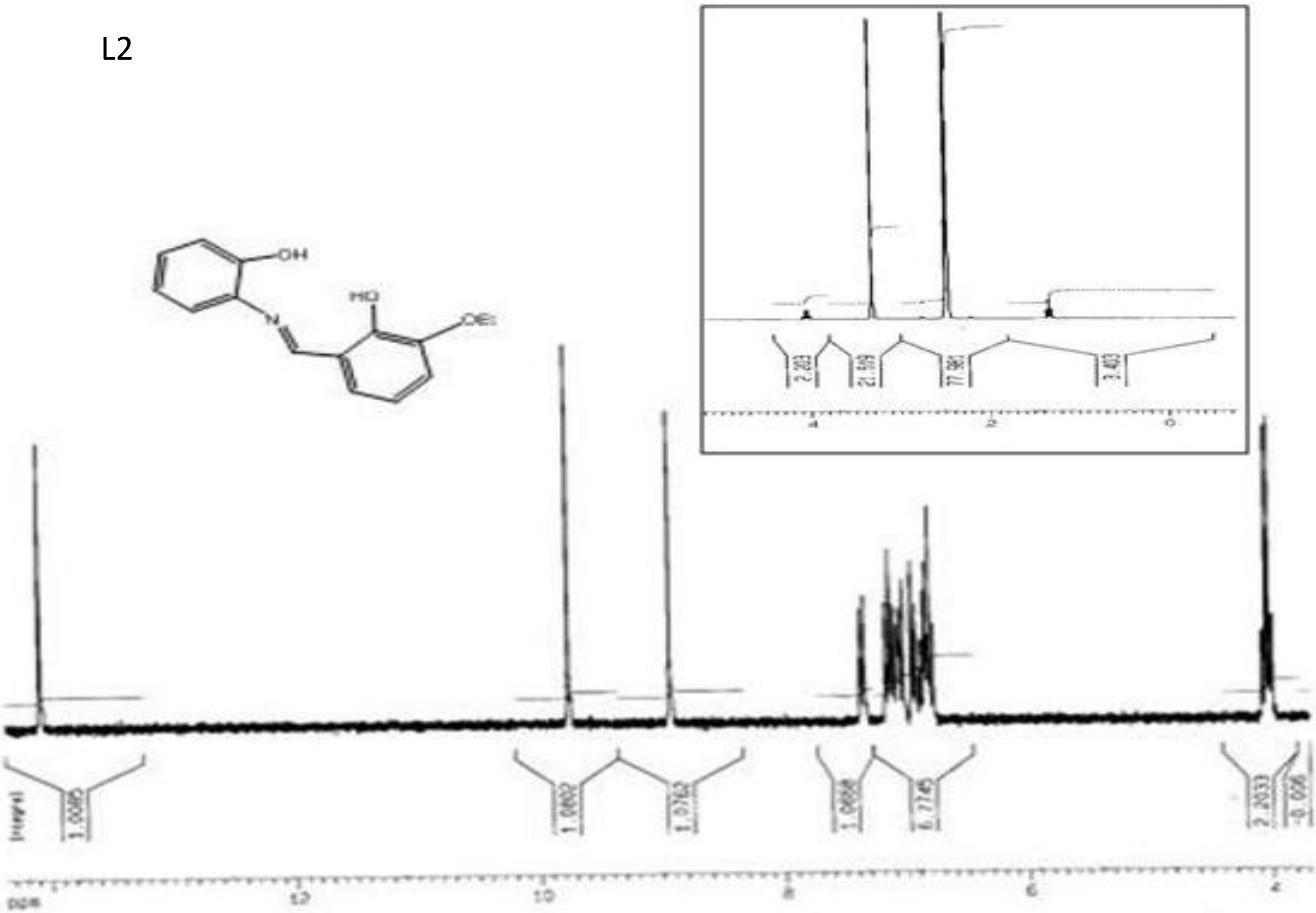
OH
HO
N
OEt
1.0085
1.0602
1.0762
1.0698
6.7745
2.2033
-0.006
ppm
12
10
8
6
4
2.203
3.403
2
0

L3

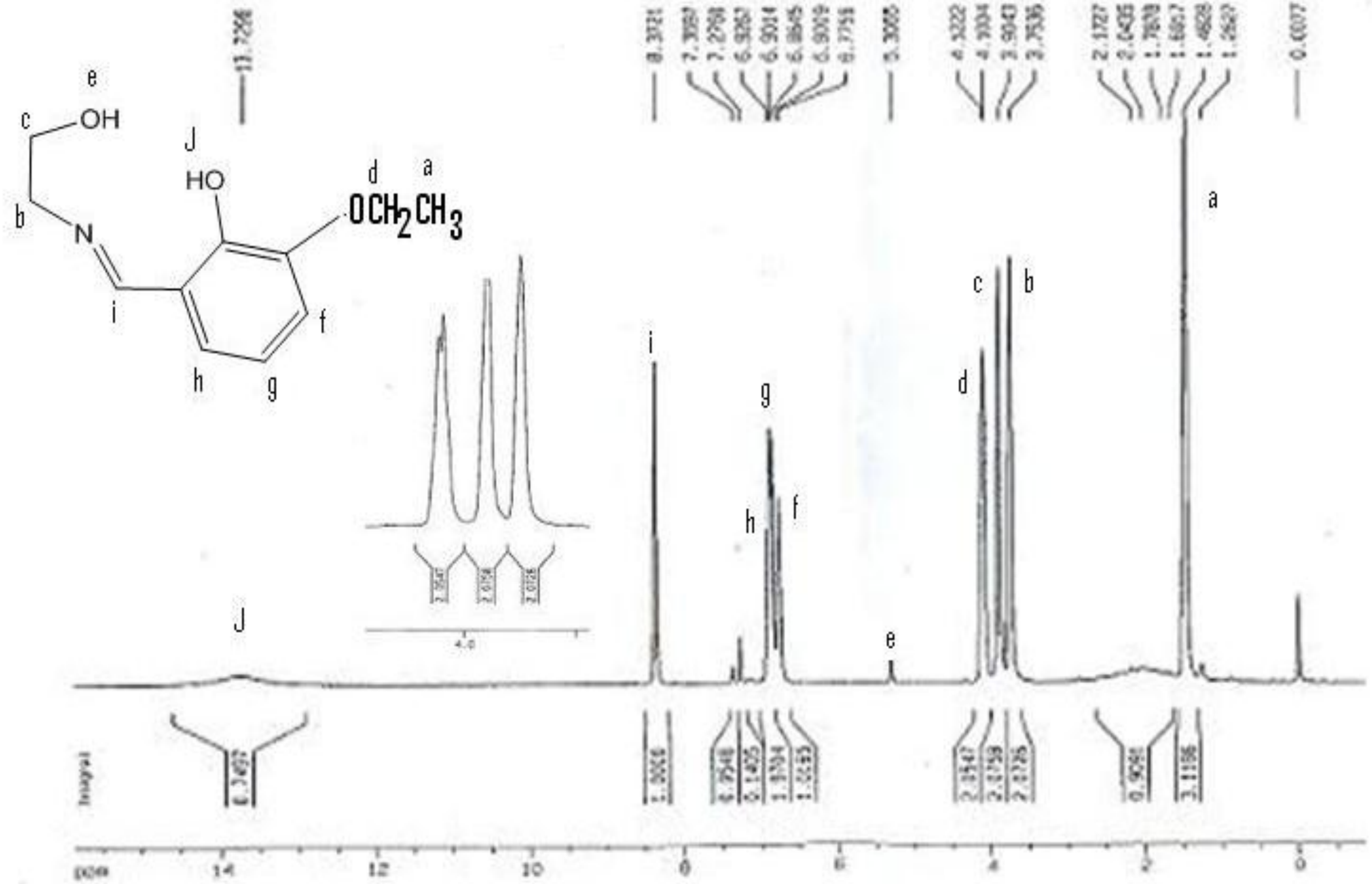

e
c
OH
J
HO
d
a
b
N
OCH2CH3
i
f
h
g
a
c
b
i
d
g
h
f
J
e
Integral
ppm
14
12
10
8
6
4
2
0

L4

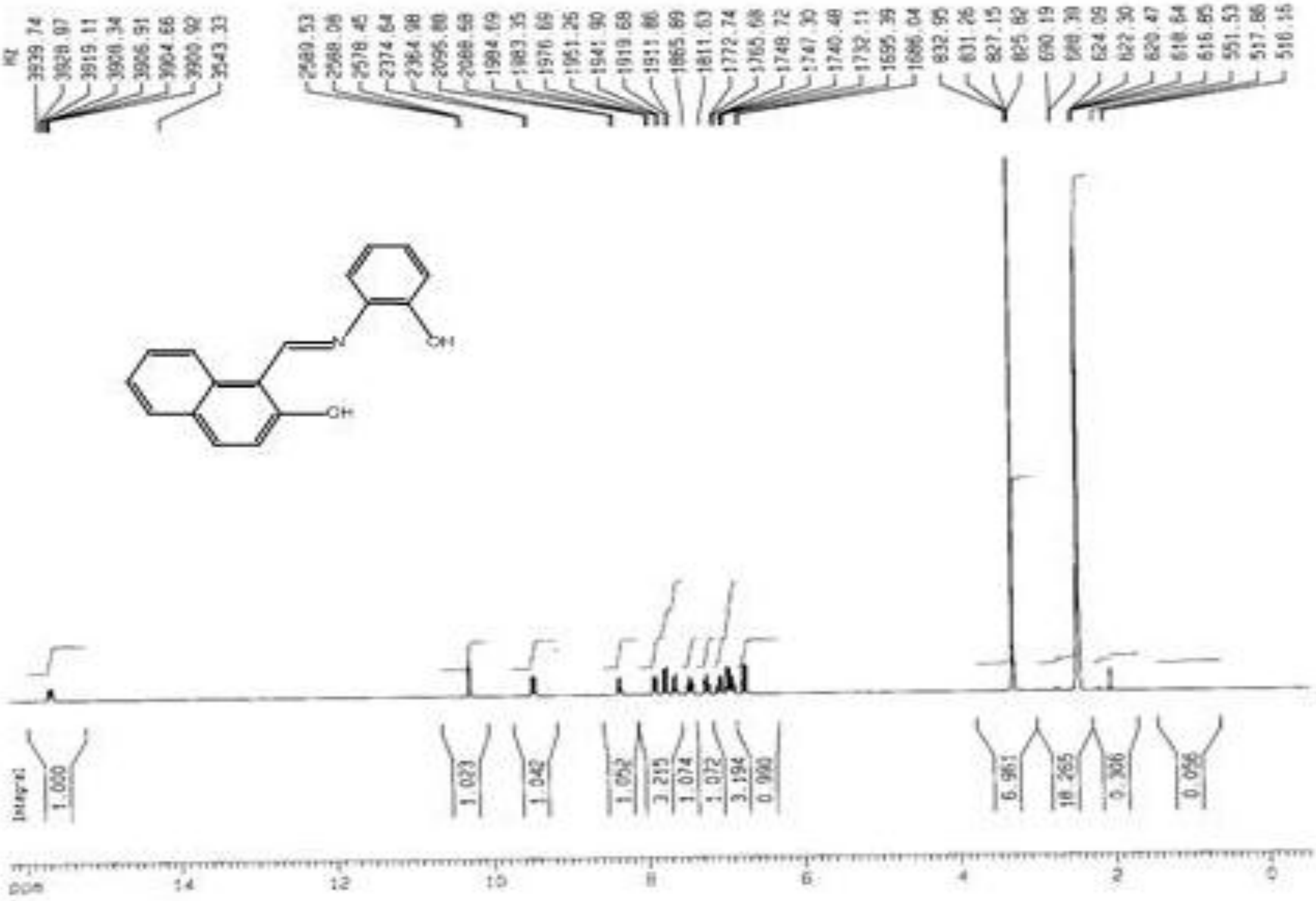
OH
OH
Integral
1.000
1.023
1.042
1.052
3.215
1.074
1.072
3.194
0.990
6.961
0.306
0.056
ppm
14
12
10
8
6
4
2
0

L1

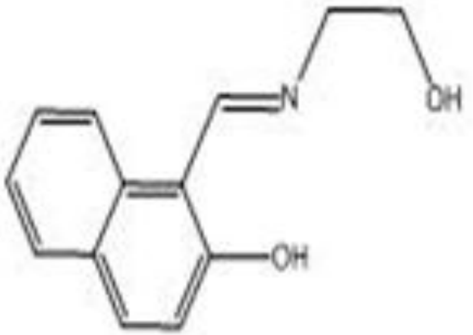
N
OH
OH

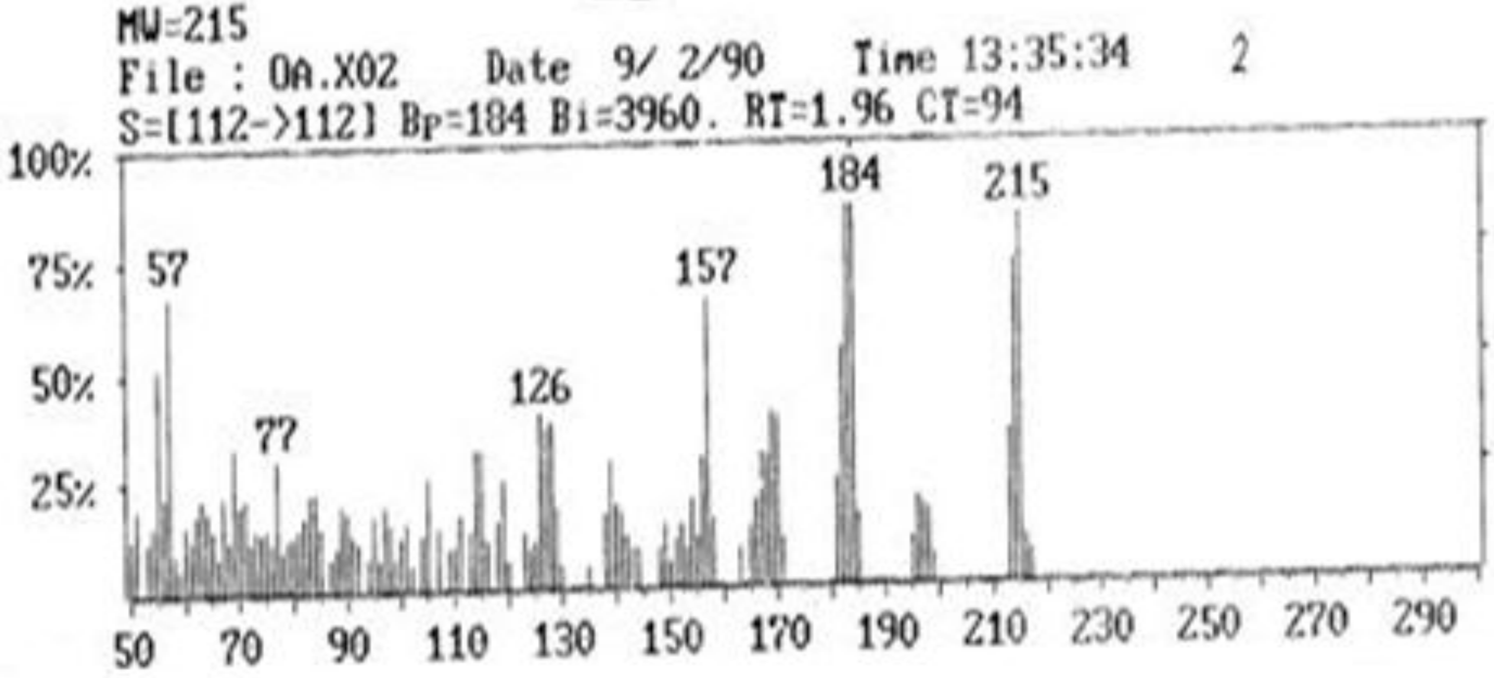
MW=215
File : OA.X02 Date 9/ 2/90 Time 13:35:34 2
S=[112->112] Bp=184 Bi=3960. RT=1.96 CT=94
100%
75%
50%
25%
57
77
126
157
184
215
50 70 90 110 130 150 170 190 210 230 250 270 290

L2

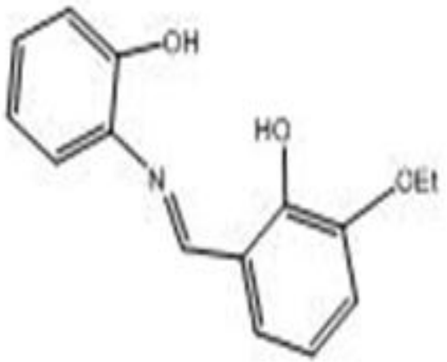

OH
HO
N
OEt

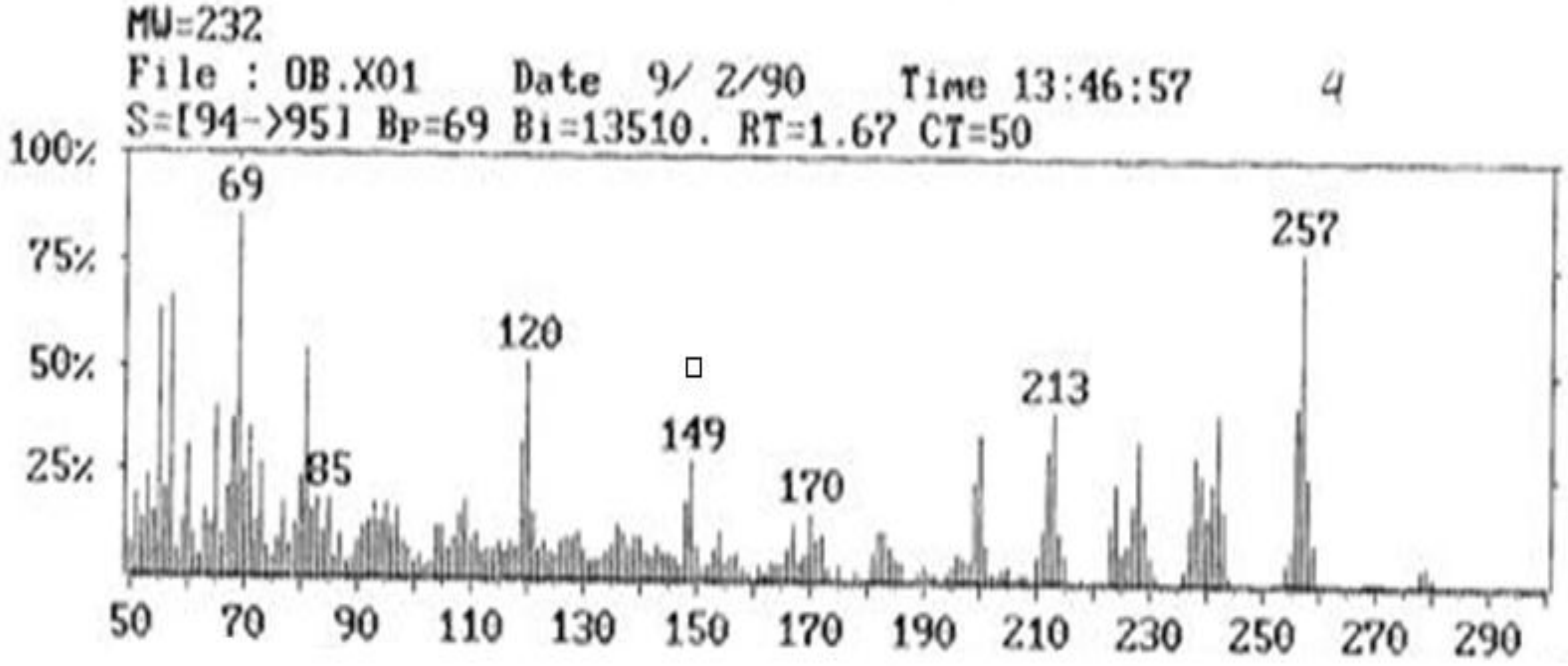

MW=232
File : OB.X01 Date 9/ 2/90 Time 13:46:57 4
S=[94->95] Bp=69 Bi=13510. RT=1.67 CT=50
100%
75%
50%
25%
69
257
120
213
149
85
170
50 70 90 110 130 150 170 190 210 230 250 270 290

L3

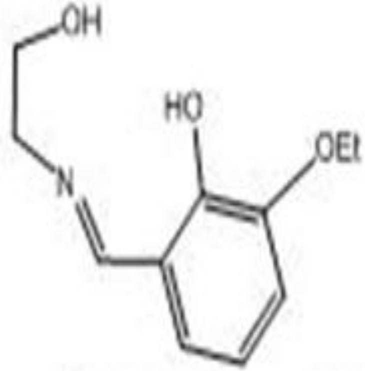
OH
HO
OEt
N

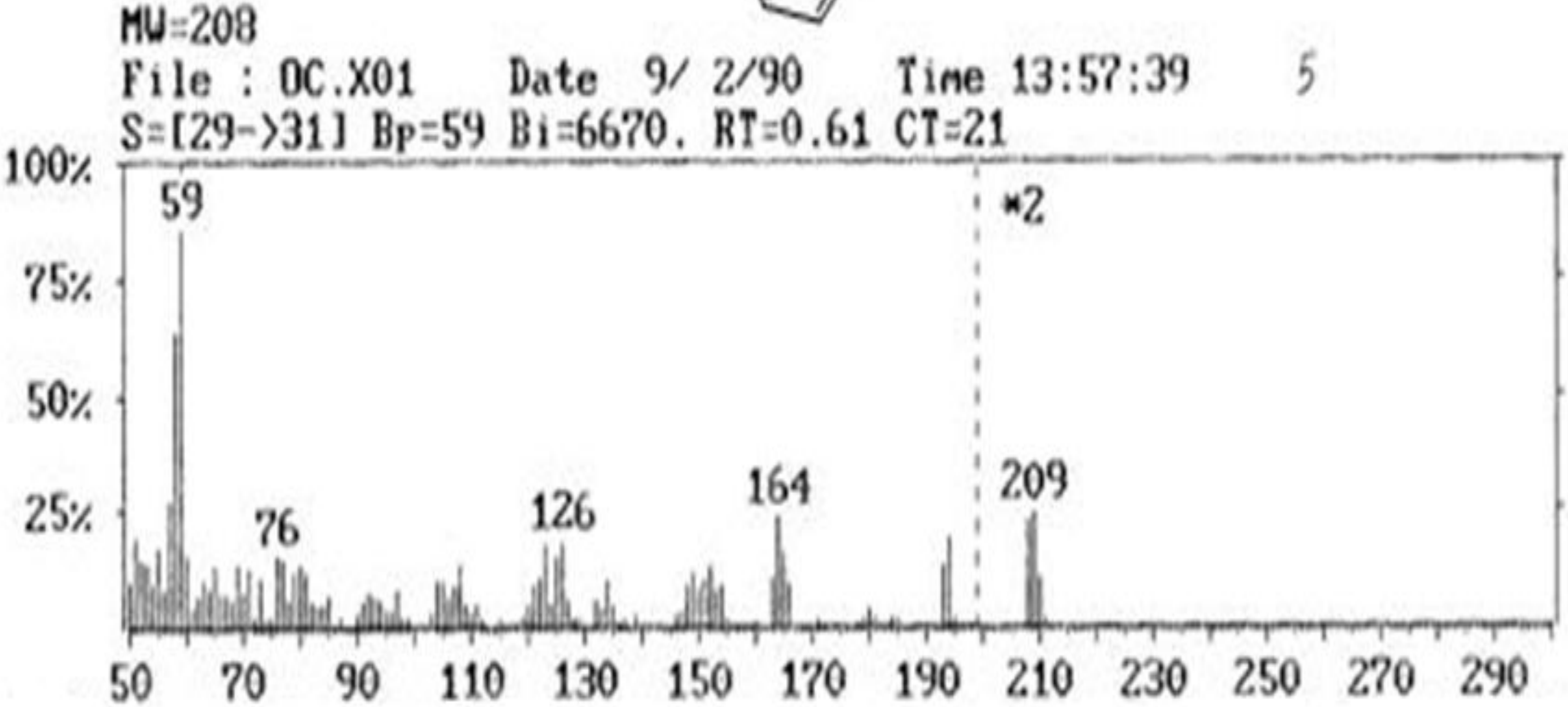
MW=208
File : OC.X01 Date 9/ 2/90 Time 13:57:39 5
S=[29->31] Bp=59 Bi=6670. RT=0.61 CT=21
100%
75%
50%
25%
59
76
126
164
209
*2
50
70
90
110
130
150
170
190
210
230
250
270
290

L4

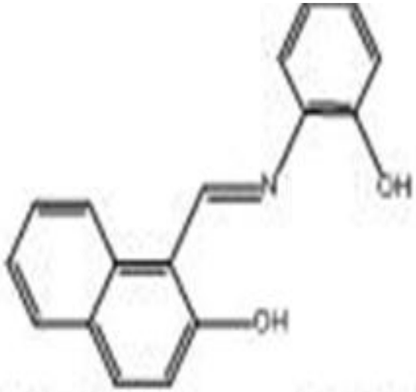

OH
OH
N

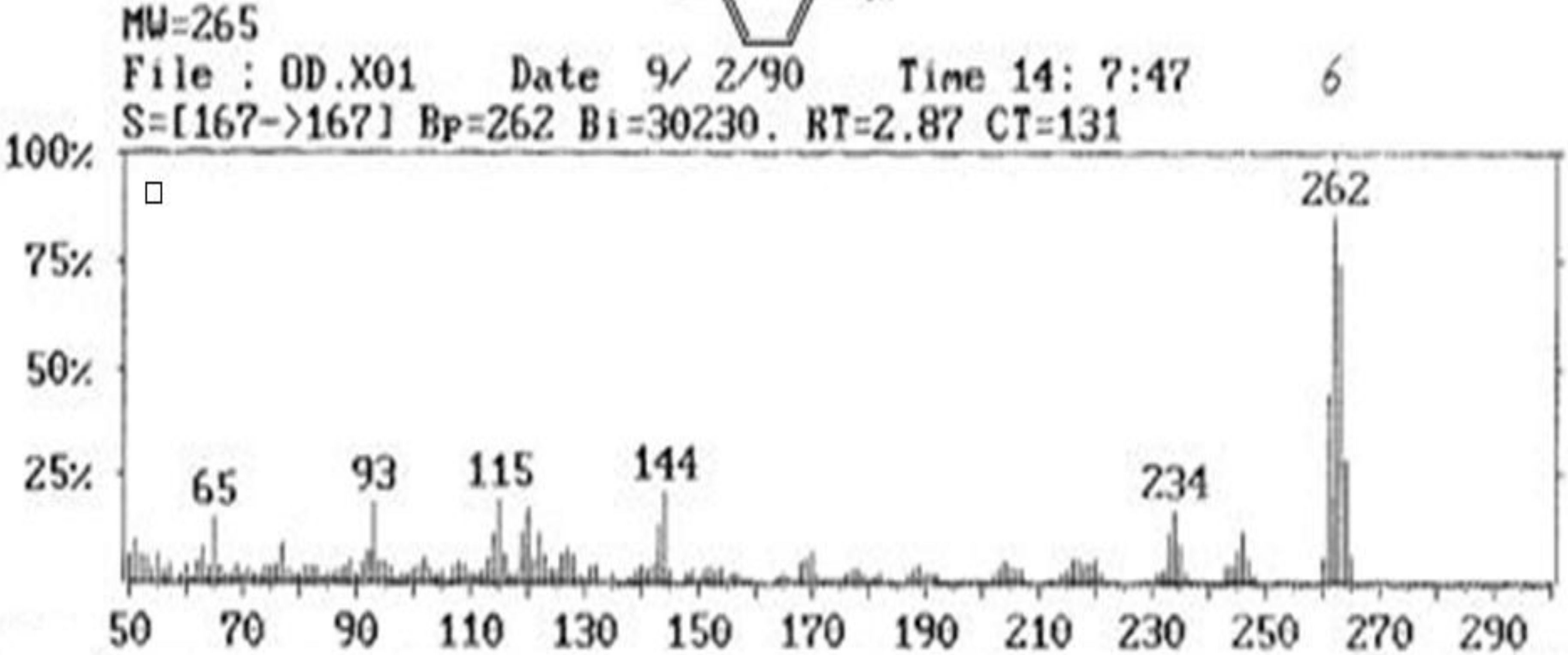

MW=265
File : OD.X01 Date 9/ 2/90 Time 14: 7:47 6
S=[167->167] Bp=262 Bi=30230. RT=2.87 CT=131
100%
75%
50%
25%
262
65
93
115
144
234
50 70 90 110 130 150 170 190 210 230 250 270 290
